# Amaryllidaceae Alkaloids of Different Structural Types from *Narcissus* L. cv. Professor Einstein and Their Cytotoxic Activity

**DOI:** 10.3390/plants9020137

**Published:** 2020-01-22

**Authors:** Kateřina Breiterová, Darja Koutová, Jana Maříková, Radim Havelek, Jiří Kuneš, Martina Majorošová, Lubomír Opletal, Anna Hošťálková, Jaroslav Jenčo, Martina Řezáčová, Lucie Cahlíková

**Affiliations:** 1 ADINACO Research Group, Department of Pharmaceutical Botany, Faculty of Pharmacy, Charles University, Heyrovského 1203, 500 05 Hradec Králové, Czech Republic; breiterk@faf.cuni.cz (K.B.); opletal@faf.cuni.cz (L.O.); HOSTA4AA@faf.cuni.cz (A.H.); jencoj@faf.cuni.cz (J.J.); 2 Department of Medical Biochemistry, Faculty of Medicine in Hradec Králové, Charles University, Šimkova 870, 500 03 Hradec Králové, Czech Republic; koutova.darja@lfhk.cuni.cz (D.K.); havelekr@lfhk.cuni.cz (R.H.); SeifrtovaM@lfhk.cuni.cz (M.M.);; 3 Department of Organic and Bioorganic Chemistry, Faculty of Pharmacy, Charles University, Heyrovského 1203, 500 05 Hradec Králové, Czech Republic; marikoj2@faf.cuni.cz (J.M.); kunes@faf.cuni.cz (J.K.)

**Keywords:** *Narcissus* cv. Professor Einstein, Amaryllidaceae, cytotoxicity, 7-oxonorpluviine, pancracine

## Abstract

In this detailed phytochemical study of *Narcissus* cv. Professor Einstein, we isolated 23 previously known Amaryllidaceae alkaloids (**1**–**23**) of several structural types and one previously undescribed alkaloid, 7-oxonorpluviine. The chemical structures were identified by various spectroscopic methods (GC-MS, LC-MS, 1D, and 2D NMR spectroscopy) and were compared with literature data. Alkaloids which had not previously been isolated and studied for cytotoxicity before and which were obtained in sufficient amounts were assayed for their cytotoxic activity on a panel of human cancer cell lines of different histotype. Above that, MRC-5 human fibroblasts were used as a control noncancerous cell line to determine the general toxicity of the tested compounds. The cytotoxicity of the tested alkaloids was evaluated using the WST-1 metabolic activity assay. The growth of all studied cancer cell lines was inhibited by pancracine (montanine-type alkaloid), with IC_50_ values which were in the range of 2.20 to 5.15 µM.

## 1. Introduction

Plants from the Amaryllidaceae family have been used for centuries in folk medicine due to their therapeutic properties [1]. Plants from this family contain a distinct and still not fully explored group of alkaloids called Amaryllidaceae alkaloids (AA). They are most well known for their broad spectrum of biological properties such as antitumor [2,3], antimalarial [4], anti-inflammatory [5], antimicrobial [6], and AChE-inhibiting activities [7,8]. The AA are classified into 9 main structural types (crinine, galanthamine, haemanthamine, homolycorine, lycorine, montanine, narciclasine, norbelladine, and tazettine) alongside more than 10 others (plicamine, galanthindole, augustamine, graciline, etc.). These minor structure types are usually found in trace amounts and are most often represented by single alkaloids [9,10]. Galanthamine, a selective, competitive acetylcholinesterase inhibitor with antioxidant properties is the most important AA identified so far. It was isolated for the first time from *Galanthus woronowii* in the 1950s. Since 2000, when it was approved, it has been used to treat mild and moderate stages of Alzheimer’s disease [11]. The ability of AA to reduce proliferative activity and viability of cancer cells is equally important. Numerous products of natural origin are known for their cytotoxic activity. These products are also a source of substances capable of affecting apoptosis resistance [12]. Simultaneously, the anti-invasive and anti-metastatic effects of some plant alkaloids have been described [13,14]. For this reason, substances of natural origin represent a valuable source of potential anticancer drugs; many AAs are very promising anticancer compounds, for example, the widely studied haemanthamine [15] and lycorine [16]. A well thought-out basic study of natural compounds can serve as a springboard for clinical therapy.

The genus *Narcissus* L. belongs to the monocotyledonous family Amaryllidaceae. Various species of this genus are commonly known as daffodil, narcissus, jonquil, etc. Genus *Narcissus* comprises about 100 wildlife species native mainly to the Mediterranean area [17]. *Narcissus* spp. have been long-established in traditional treatment of several cancer illnesses throughout the world. Hippocrates of Kos (460–370 B.C.), the famous Ancient Greek physician, recommended a pessary impregnated with narcissus oil to treat uterine tumors [18]. The application of narcissus oil in cancer treatment was also recorded in the Middle Ages in China, North Africa, Central America, and Arabian countries [19].

Due to the ease of natural hybridization, spreading, and naturalization, as well as extensive cultivation, the taxonomy of *Narcissus* spp. is quite complicated. A broad spectrum of cultivars has been bred mainly for ornamental purposes, and the International Daffodil Register contains over 27,000 names of these cultivars [20]. For commercial production of AAs, the cultivars are preferred over native species because of their availability. Some *Narcissus* cultivars have been already studied in detail, and a number of AAs have been isolated throughout the years [7,10,21]. From previous phytochemical studies of 40 differing *Narcissus* cultivars, 14 AAs were identified in the alkaloid extract of *Narcissus* cv. Professor Einstein [17] (see Supplementary Materials). The alkaloid extract also showed promising cytotoxic activity against different cancer cell types in a preliminary study (see section Results). The interesting bioactivities and lack of detailed phytochemical studies of *N*. cv. Professor Einstein led us to explore the properties and alkaloid content of this cultivar.

## 2. Results

### 2.1. Isolation and Identification of Amaryllidaceae Alkaloids from N. cv. Professor Einstein

**Figure 1:**
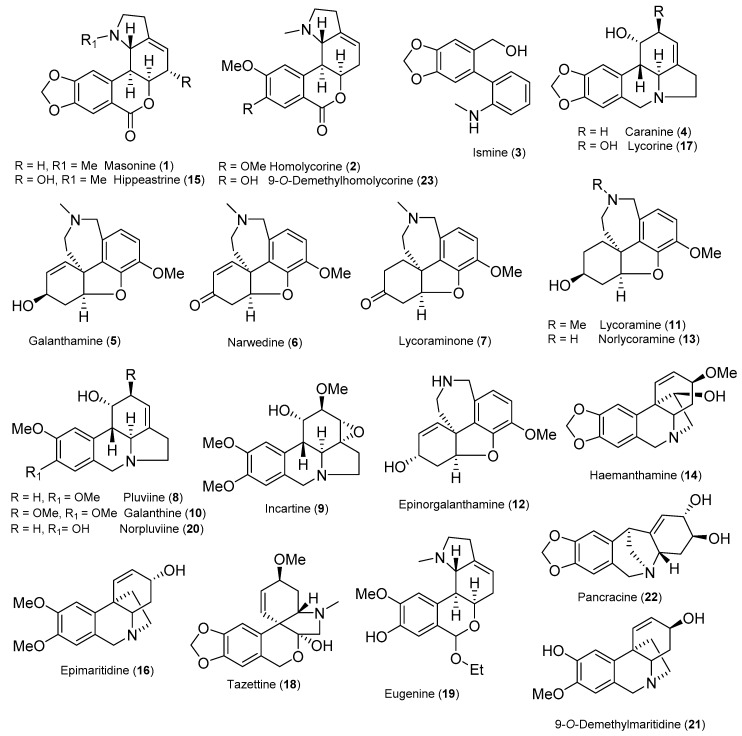
Structures of isolated alkaloids.

Extensive phytochemical study of fresh bulbs of *N.* cv. Professor Einstein resulted in the isolation of 23 known AA (**1**–**23**), and a new alkaloid (**24**). The compounds were analyzed by various spectroscopic methods (GC-MS, LC-MS, and 1D and 2D NMR spectroscopy) and identified by comparison with literature data as masonine (**1**) [22], homolycorine (**2**) [23], ismine (**3**) [24], caranine (**4**) [25], galanthamine (**5**) [26], narwedine (**6**) [27], lycoraminone (**7**) [28], pluviine (**8**) [25], incartine (**9**) [29], galanthine (**10**) [30], lycoramine (**11**) [26], epinorgalanthamine (**12**) [31], norlycoramine (**13**) [32], haemanthamine (**14**) [32], hippeastrine (**15**) [33], epimaritidine (**16**) [34], lycorine (**17**) [35], tazettine (**18**) [36], eugenine (**19**) [37], norpluviine (**20**) [38], 9-*O*-demethylmaritidine (**21**) [39], pancracine (**22**) [40], and 9-*O*-demethylhomolycorine (**23**) [23] (Figure 1). The alkaloids that were isolated were representatives of the homolycorine (**1**, **2**, **15**, **19**, **23**), galanthamine (**5**, **6**, **7**, **11**, **12**, **13**), haemanthamine (**14**, **16**, **21**), lycorine (**4**, **8**, **9**, **10**, **17**, **20**), montanine (**22**), tazettine (**18**), and miscellaneous (**3**) structure types.

**Figure 2:**
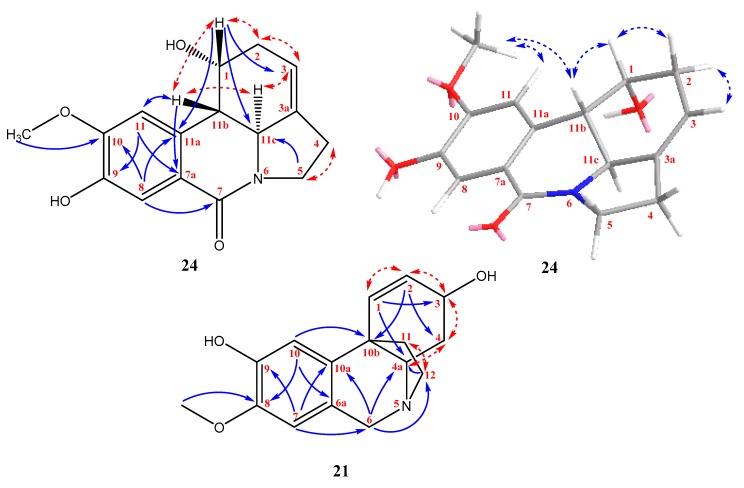
The constitution of **21**; the constitution of **24** and its relative configuration. The key gCOSY (red dashed arrows), gHMBCAD (blue arrows), and NOESY (blue dashed arrows) correlations are marked.

The structure of compound **21** is already known, but confirmation of it was not possible due to the absence of its NMR data in the available literature. This led us to publish the complete ^1^H and ^13^C NMR spectra for this alkaloid. The structural constitution was elucidated utilizing 2D experiments such as gCOSY, gHSQC, and gHMBCAD (see Figure 2).

The new lycorine-type Amaryllidaceae alkaloid **24**, named 7-oxonorpluviine, was isolated as a white amorphous solid. Its HR-MS data showed a molecular ion [M + H]^+^ at *m*/*z* 288.1242, corresponding to the formula C_16_H_17_NO_4_. The ^1^H NMR data of **24** exhibited the presence of two aromatic protons (δ_H_ 7.45, H-8; 6.99, H-11), one olefinic proton (δ_H_ 5.71–5.67, H-3), one deshielded hydroxymethine proton (δ_H_ 4.95–4.91, H-1), one methoxy group (δ_H_ 3.98, C-10-OCH_3_), one nitrogenated methine (δ_H_ 3.53–3.47, H-11c), one deshielded methylene group with diastereotopic protons (δ_H_ 3.35–3.25 and 3.06–2.98, H-5), one methine proton (δ_H_ 2.84, H-11b), and two methylene groups (δ_H_ 2.79–2.62 and 2.61–2.54, H-2 and H-4) (Table 1). While two protons were not detected by NMR in methanol-*d*_4_, they should have been bound to the heteroatoms. The ^13^C NMR spectrum exhibited 16 carbons. A 1,2,4,5-tetrasubstituted benzene ring was determined by gHMBCAD experiment. The identification was based on correlations from H-11 to C-7a (δ_C_ 117.4) and C-9 (δ_C_ 148.1), and from H-8 to C-10 (δ_C_ 154.5) and C-11a (δ_C_ 136.5), respectively. The deshielded aromatic carbons were substituted by a hydroxyl group (C-9) and a methoxy group (C-10). The carbonyl group C-7 (δ_C_ 167.6) was identified in the ^13^C NMR spectrum; this signal was shown to be correlated with H-8 in gHMBCAD (Figure 2). The tertiary carbon C-11b (δ_C_ 41.9), which was correlated to H-11, was determined as a junction point between the aromatic substructural fragment and the rest of the molecule. The gCOSY experiment of **24** revealed cross-peaks of H-11b with H-11c and H-1, H-2 with H-1, H-3 with H-2 and H-11c, and H-5 with H-4. These correlations determined the hexahydroindole fragment in the structure (Figure 2). In addition, the gHMBCAD experiment supported this assumption by correlations from H-1 to C-3 (δ_C_ 118.3), from H-1 to C-11c (δ_C_ 60.9), from H-1 to C-11a (δ_C_ 137.7), and from H-5 to C-11c. The absolute configuration could not be determined due to insufficient amount of sample. The relative configuration was established and the result was supported by NOESY experiment (see Figure 2). This suggested configuration of either 1*R*, 11b*S*, 11c*S* or 1*S*, 11b*R*, 11c*R* is supported by the cross-peaks of H-11b and H-1. Moreover, no correlation of these protons with H-11c was detected.

### 2.2. Cytotoxic Study of Isolated Amaryllidaceae Alkaloids

A set of cell lines derived from various human tumor histotypes (leukemia—Jurkat, MOLT-4; adenocarcinomas of lung—A549, colon—HT-29, pancreas—PANC-1, cervix—HeLa, breast—MCF-7; ovarian carcinoma—A2780, and osteogenic sarcoma—SAOS-2) was used to screen the cytotoxic activity of all isolated compounds which had not been previously studied and were obtained in sufficient quantities. Normal human fetal lung fibroblasts (MRC-5) were used to study effect on non-tumorigenic cells. Growth-inhibitory activity of tested alkaloids was determined at a dose of 10 µM (Table 2) using the WST-1 mitochondrial dehydrogenase activity assay.

The efficacy of each alkaloid in decreasing the growth of individual cell line, referred as the mean growth percentage (GP), was calculated as an average of all cell lines proliferation in percent. The threshold GP value for this screen was <50% (50% tumor growth inhibition), meaning good activity at 10 µM, as can be seen in the result for pancracine (**22**) in Table 2. Pancracine was able to inhibit all cancer cells used in the study, with the exception of PANC-1, where IC_50_ values ranged from 2.20 to 5.15 µM (Table 3).

**Figure 3:**
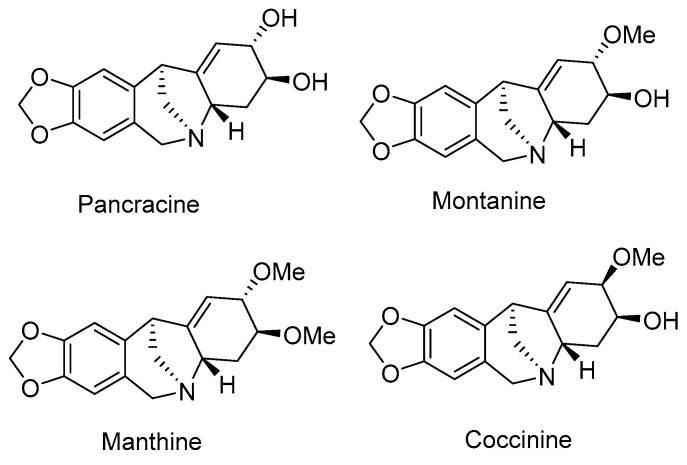
Structures of selected montanine-type Amaryllidaceae alkaloids.

Pancracine (**22**) is a montanine type of AA, in the same group as montanine, coccinine, and manthine. This structural type of AA seems to be a promising souce of compounds in the search for new anticancer drugs. They are characterized by a 5,11-methanomorphantridine ring system, the only differencies being the substitutions and configuration at C-2 and C-3 centers (Figure 3) [40]. Some of these compounds have been screened against different cancerous cells [12,41]. 

Montanine and manthine showed strong in vitro growth inhibitory effect on three apoptosis-resistant cancer cell lines (A549, SKMEL-29, and U373) and three apoptosis-sensitive cancer cell lines (MCF7, Hs683, and B16F10) with IC_50_ values between 5 and 31 µM [12]. In another recent study, C-2α-/C-2β-methoxy isomers montanine and coccinine were found to significantly affect the proliferation of human breast, colon, lung, and melanoma cancer cell lines over 48 h of treatment. The obtained results revealed that montanine has a more promising cytotoxic activity (IC_50_ values were 1.9 ± 0.4 µM for A549 cells, 6.8 ± 0.5 µM for HCT-15 cells, 23.2 ± 1.9 µM for SK-MEL-28 cells, 4.4 ± 0.4 µM for MCF-7 cells, and 3.4 ± 0.9 µM for MDA-MB-231 cells, 3.6 ± 1.7 µM for Hs578T cells) when compared with coccinine (IC_50_ values were 5.9 ± 0.8 µM for A549 cells, 16.8 ± 1.8 µM for HCT-15 cells, >50 µM for SK-MEL-28 cells, 7.9 ± 0.9 µM for MCF-7 cells, 13.8 ± 0.8 µM for MDA-MB-231 cells, and 5.3 ± 0.4 µM for Hs578T cells) [42]. Although previous studies, as well as our work, have demonstrated that AA of the montanine type can effectively suppress viability and proliferation of human cancer cells, the molecular mechanism of this cytotoxic activity has not yet been fully explored and is still waiting to be described.

## 3. Experimental

### 3.1. General Experimental Procedures

All solvents were treated using standard techniques before use. All reagents were purchased from commercial sources (Sigma Aldrich, Prague, Czech Republic) and used without purification. The NMR spectra were obtained in CDCl_3_ and CD_3_OD at ambient temperature on a VNMR S500 (Varian, Palo Alto, CA, USA) spectrometer operating at 500 MHz for ^1^H and 125.7 MHz for ^13^C. Chemical shifts were recorded as *δ* values in parts per million (ppm) and were indirectly referenced to tetramethylsilane (TMS) via the solvent signal (CDCl_3_—7.26 ppm for ^1^H and 77.0 ppm for ^13^C; CD_3_OD—3.30 ppm for ^1^H and 49.0 ppm for ^13^C). Coupling constants (*J*) are given in Hz. For unambiguous assignment of ^1^H and ^13^C signals, 2D NMR experiments, namely gCOSY, gHSQC, gHMBC, and NOESY were measured using standard parameter settings and standard pulse programs provided by the producer of the spectrometer. HRMS were obtained with a Waters Synapt G2-Si hybrid mass analyzer of a quadrupole-time-of-flight (Q-TOF) type, coupled to a Waters Acquity I-Class UHPLC system. The EI-MS were obtained on an Agilent 7890A GC 5975 inert MSD operating in EI mode at 70 eV (Agilent Technologies, Santa Clara, CA, USA). A DB-5 column (30 m × 0.25 mm × 0.25 μm, Agilent Technologies, USA) was used. The temperature program was: 100–180 °C at 15 °C/min, 1 min hold at 180 °C, 180–300 °C at 5 °C /min, and 5 min hold at 300 °C; detection range *m*/*z* 40–600. The injector temperature was 280 °C. The flow-rate of the carrier gas (helium) was 0.8 mL/min. A split ratio of 1:15 was used. TLC was carried out on Merck precoated silica gel 60 F254 plates. Compounds on the plate were observed under UV light (254 and 366 nm) and visualized by spraying with Dragendorff’s reagent.

### 3.2. Plant Material

Fresh bulbs of *Narcissus* cv. Professor Einstein were obtained from the herbal dealer Lukon Glads (Sadská, Czech Republic). The botanical identification was performed by Professor L. Opletal, CSc. A voucher specimen is deposited in the Herbarium of the Faculty of Pharmacy in Hradec Králové under number CUFPH-16130/AL-447.

### 3.3. Extraction and Isolation of Alkaloids

Fresh bulbs (34 kg) were cut, minced, and underwent comprehensive extraction with ethanol (EtOH) (96%, *v*/*v*, 2×). Each portion (approximately 1.2 kg) was boiled twice for 30 min under reflux. The combined extracts were filtered and evaporated under reduced pressure. The crude extract (638 g) was dissolved in 1.5 L of 5% hydrochloric acid (HCl), filtered twice, and diluted with distilled water to 4.5 L at pH 1. The water solution was defatted with diethyl ether (Et_2_O; 2 × 4 L), alkalized with 10% Na_2_CO_3_ to pH 10, and exhaustively extracted with chloroform (CHCl_3_; 4 × 4 L). The organic phase was evaporated to give 58 g of dark brown residue. The purification process was repeated to give 32 g of concentrated alkaloidal residue (brown syrup). The obtained extract, which was Dragendorff positive, was fractionated by column chromatography on neutral Al_2_O_3_ (ACROSS, 2100 g) deactivated with 6 % of distilled water. The extract was eluted with light petrol enriched with CHCl_3_ (gradually from 30:70 to 80:20), then with CHCl_3_ gradually enriched with EtOH (from 1:99 to 50:50), and finally with pure EtOH. Fractions of 250 mL were collected and monitored by TLC, yielding 495 fractions, which were combined into 15 final fractions and analyzed by TLC and GC-MS.

Combined fractions **I**–**III** (60 mg) were separated by preparative TLC (toluene:EtOAc: Et_2_NH 8:1:1, 1×) and gave three sub-fractions. Sub-fraction **I**–**III/2** was recrystalized from an ethanol and chloroform mixture (1:1) to give masonine (**1**, 5 mg). Sub-fraction **I**–**III/3** was further chromatographed by preparative TLC (toluene:Et_2_NH 95:5, 1×) to give homolycorine (**2**, 6 mg). Fractions **IV** (35 mg) and **VI** (25 mg), based on TLC, contained no alkaloidal compound, and were not used for isolation of alkaloids. Preparative TLC of fraction **V** (50 mg; toluene:EtOAc:Et_2_NH 8:1:1, 1×) gave sub-fractions **V/1**–**V/4**. Sub-fraction **V/3** was further chromatographed by preparative TLC (cyclohexane:Et_2_NH 9:1, 2×) to give ismine (**3**, 4 mg). Fraction **VII** (1.69 g) was chromatographed by preparative TLC (toluene:Et_2_NH 95:5, 2×) to give sub-fractions **VII/1**–**VII/4**. Caranine (**4**, 7 mg) was obtained by subsequent TLC of sub-fraction **VII/2** (201 mg). Sub-fraction **VII/3** (350 mg) was subjected to repeated preparative TLC (cyclohexane: toluene:Et_2_NH 50:45:5, 2×) to give galanthamine (**5**, 35 mg), narwedine (**6**, 15 mg), and lycoraminone (**7**, 30 mg). Pluviine (**8**, 25 mg) was obtained from sub-fraction **VII/4** (150 mg) by crystallization from hot ethanol (10 mL). Fraction **VIII** (30 mg) was subjected to preparative TLC (cyclohexane:isopropanol:Et_2_NH 77.5:15:7.5, 1×) to give sub-fractions **VIII/1**–**VIII/4**. Sub-fraction **VIII/2** (203 mg) was further chromatographed by preparative TLC (cyclohexane:Et_2_NH 95:5, 2×) to give incartine (**9**, 5 mg), galanthine (**10**, 6 mg), and lycoramine (**11**, 80 mg). Epinorgalanthamine (**12**, 10 mg) was obtained from **VIII/4** by preparative TLC (cyclohexane:EtOAc:isopropanol:Et_2_NH 45:45:5:5, 2×). Fraction **IX** (138 mg) was chromatographed by preparative TLC (toluene:EtOAc:Et_2_NH 7:2.5:0.5, 1×) to give norlycoramine (**13**, 35 mg). Haemanthamine (**14**, 320 mg) was obtained from fraction **X** (1.02 g) by crystallization from an ethanol and chloroform mixture (1:1). Hippeastrine (**15**, 166 mg) was obtained from fraction **XI** (616 mg) by crystallization from an ethanol and chloroform mixture (1:1). Mother liquor of fraction **XI** was subjected to preparative TLC (toluene:EtOAc:Et2NH 15:75:10, 1×) to give epimaritidine (**16**, 127 mg). Fraction **XII** (1.43 g) was dissolved in hot ethanol and lycorine (**17**, 752 mg) crystallized from the mixture. Tazzetine (**18**, 1.85 g) crystallized (EtOH:CHCl_3_ 7:3, 100 mL) from fraction **XIII** (3.11 g). The mother liquor (890 mg) was chromatographed by preparative TLC (cyclohexane:acetone:NH_3_ 50:50:1, 3×) to give sub-fractions **XIII/1**–**XIII/3**. Subfraction **XIII/1** (89 mg) was dissolved in hot ethanol and eugenine (**19**, 20 mg) crystallized from the solution. Fraction **XIV** (6.5 g), by column chromatography on Al_2_O_3_ (800 g), yielded seven sub-fractions **XIV/1**–**XIV/7**. Subfraction **XIV/6** was subjected to preparative TLC (cyclohexane:acetone:NH_3_ 10:80:1) to give norpluviine (**20**, 62 mg) and 9-*O*-demethylmaritidine (**21**, 15 mg). Subfraction **XIV/7** (2.5 g) was subjected to preparative TLC (toluene:acetone:EtOH:NH_3_ 40:40:6:4, 2×) to obtain pancracine (**22**, 247 mg) and subfraction **XIV/7-B** (1.95 g). 9-*O*-Demethylhomolycorine (**23**, 1.42 g) was obtained from sub-fraction **XIV/7-B** by preparative TLC (toluene:Et_2_NH, 95:5, 3×). Fraction **XV** (672 mg) was separated by preparative TLC (EtOAc:MeOH:Et_2_NH 8:1:1, 1×) into four sub-fractions, **XV/1**–**XV/4**. Sub-fraction **XV/3** was further chromatographed by preparative TLC to give 7-oxonorpluviine (**24**, 6 mg).

### 3.4. In Vitro Cytotoxicity Study

#### 3.4.1. Cell Culture and Culture Conditions

Selected human tumor and non-tumor cell lines Jurkat (acute T cell leukemia), MOLT-4 (acute lymphoblastic leukemia), A549 (lung carcinoma), HT-29 (colorectal adenocarcinoma), PANC-1 (pancreas epithelioid carcinoma), A2780 (ovarian carcinoma), HeLa (cervix adenocarcinoma), MCF-7 (breast adenocarcinoma), SAOS-2 (osteosarcoma), and MRC-5 (normal lung fibroblasts) were purchased from either ATCC (Manassas, VA, USA) or Sigma-Aldrich (St. Louis, MO, USA) and cultured according to the providers‘ culture method guidelines. All cell lines were maintained at 37 °C in a humidified 5% carbon dioxide and 95% air incubator. Cells in the maximum range of either 10 passages for a primary cell line (MRC-5), or in the maximum range of 20 passages for cancer cell lines (Jurkat, MOLT-4, A549, HT-29, PANC-1, A2780, HeLa, MCF-7, and SAOS-2) and in an exponential growth phase were used for this study.

#### 3.4.2. Cell Treatment

All the alkaloids evaluated and doxorubicin, used as positive control, were dissolved in dimethyl sulfoxide (DMSO) (Sigma-Aldrich, St. Louis, MO, USA) to prepare stock solutions with a concentration of 10–50 mM based on their solubility. Stock solutions were freshly prepared before use in the experiments. For the experiments, the stock solutions were diluted with the appropriate culture medium to create final concentrations (10 µM for a single-dose alkaloid cytotoxicity screen and 1 µM for doxorubicin, used as a reference compound) making sure that the concentration of DMSO was <0.1% to avoid toxic effects on the cells. Control cells were sham-treated with a DMSO vehicle only (0.1%; control).

#### 3.4.3. WST-1 Cytotoxicity Assay

The WST-1 (Roche, Mannheim, Germany) reagent was used to determine the cytostatic effect of the test compounds. WST-1 is designed for the spectrophotometric quantification of cell proliferation, growth, viability, and chemosensitivity in cell populations using a 96 well plate format (Sigma, St. Louis, MO, USA). The principle of WST-1 is based on photometric detection of the reduction of tetrazolium salt to a colored formazan product. The cells were seeded at a previously established optimal density (30,000 Jurkat, 25,000 MOLT-4, 500 A549, 1500 HT-29, 2000 PANC-1, 5000 A2780, 500 HeLa, 1500 MCF-7, 2000 SAOS-2, and 2000 MRC-5 cells/well) in 100 µL of culture medium, and adherent cells were allowed to reattach overnight. Thereafter, the cells were treated with 100 µL of either corresponding alkaloids or doxorubicin stock solutions to obtain the desired concentrations and incubated in 5% CO_2_ at 37 °C. WST-1 reagent diluted 4-fold with PBS (50 µL) was added 48 h after treatment. Absorbance was measured after 3 h incubation with WST-1 at 440 nm. The measurements were performed in a Tecan Infinite M200 spectrometer (Tecan Group, Männedorf, Switzerland). All experiments were performed at least three times with triplicate measurements at each drug concentration per experiment. The viability was quantified as described in Havelek et al. according to the following formula: (%) viability = (Asample − Ablank)/(Acontrol − Ablank) × 100, where A is the absorbance of the employed WST-1 formazan measured at 440 nm [43]. The viability of the treated cells was normalized to the viability of cells treated with 0.1% DMSO (Sigma-Aldrich, St. Louis, MO, USA) as a vehicle control.

### 3.5. Statistical Analysis

The descriptive statistics of the results were calculated and the charts made in either Microsoft Office Excel 2010 (Microsoft, Redmond, WA, USA) or GraphPad Prism 5 biostatistics (GraphPad Software, La Jolla, CA, USA). In this paper, all of the values have been expressed as arithmetic means with SD of triplicates (n = 3), unless otherwise noted. The significant differences between the groups were analyzed using Student’s t-test and a *p* value ≤ 0.05 was considered statistically significant. IC_50_ curves of pancracine and standard cytostatic treatment doxorubicin were generated and values of IC_50_ were deducted with GraphPad Prism 5 Biostatistics.

## 4. Conclusions

In conclusion, 24 Amaryllidaceae alkaloids of various structural types were isolated from fresh bulbs of *Narcissus* cv. Professor Einstein. The constitution of a newly isolated alkaloid, 7-oxopluviine, was structurally determined. The complete NMR data for 9-*O*-demethylmaritidine are reported. Isolated compounds not previously studied for cytotoxicity were assayed for their cytotoxicity on a set of nine human cancer cells of different histotypes. This study revealed promising cytotoxic activity of pancracine, a montanine-type alkaloid, which was isolated in an amount that will allow more detailed cytotoxic studies in the future, and also the preparation of semisynthetic derivatives. Taken together, the plant cultivar *Narcissus* cv. Professor Einstein is as a rich source of diverse alkaloids, some of which show interesting activities for further pharmaceutical research.

## Figures and Tables

**Table 1:** 9-*O*-demethylmaritidine (**21**) and 7-oxonorpluvine (**24**) ^1^H and ^13^C NMR data.

Position	9-*O*-Demethylmaritidine (21) ^a^		7-Oxonorpluviine (24) ^b^	
	*δ*_C_	*δ*_H_ (*J* in Hz)	*δ*_C_	*δ*_H_ (*J* in Hz)
1	129.0	6.51 dd (10.2, 2.2)	77.3	4.95–4.91 m
2	131.1	5.79–5.74 m	32.2	2.79–2.62 m 2.61–2.54 m
3	67.8	4.48–4.41 m	118.3	5.71–5.67 m
3a	-	-	137.7	
4	34.9	2.25–2.05 m 1.66–1.53 m	29.5	2.79–2.62 m
4a	66.7	3.26 dd (13.3, 3.6)	-	-
5	-	-	44.9	3.35–3.25 m 3.06–2.98 m
6	61.8	4.48–4.41 m 3.81 d (16.9)	-	-
6a	123.9		-	-
7	109.2	6.51 s	167.6	
7a	-	-	117.4	
8	145.0		117.3	7.45 s
9	144.0		148.1	
10	108.5	6.88 s	154.5	
10a	138.2		-	-
10b	44.0		-	-
11	44.9	2.25–2.05 m	111.1	6.99 s
11a	-	-	136.5	
11b	53.2	3.50–3.42 m 2.98–2.90 m	41.9	2.84 dd (10.3, 2.2)
11c	-	-	60.9	3.53–3.47 m
C8-OCH_3_	55.9	3.84 s	-	-
C10-OCH_3_	-	-	56.7	3.98 s

^a^ measured in CDCl_3_; ^b^ measured in CD_3_OD; 500 MHz for ^1^H and 125.7 MHz for ^13^C; *δ* in ppm.

**Table 2:** Cytotoxic activity of alkaloidal extract of *N.* cv. Professor Einstein (concentration 50 µg·mL^−1^), and isolated Amaryllidaceae alkaloids **1**, **2**, **7**, **13**, **16**, **20**, **21**, **22**, and **23** using a single dose (concentration of 10 µM). WST-1 metabolic activity assay was used to analyze cell growth 48 h after treatment. The results are expressed as the mean values ± SD of at least three independent experiments (*n* = 3). Cells treated with 1 µM doxorubicin served as a positive control.

Cell Type	Extract	1	2	7	13	16	20	21	22	23	Dox.
Jurkat	3 ± 1	76 ± 5	78 ± 7	102 ± 9	102 ± 2	102 ± 4	96 ± 12	97 ± 6	17 ± 5	100 ± 10	2 ± 0
MOLT-4	3 ± 2	105 ± 8	110 ± 5	99 ± 2	106 ± 1	106 ± 4	107 ± 8	95 ± 9	1 ± 0	100 ± 3	0 ± 0
A549	25 ± 2	109 ± 22	114 ± 21	107 ± 8	93 ± 7	110 ± 10	111 ± 2	104 ± 7	29 ± 3	109 ± 9	11 ± 5
HT-29	41 ± 6	110 ± 17	199 ± 11	98 ± 7	94 ± 1	103 ± 5	92 ± 5	89 ± 10	39 ± 3	106 ± 8	47 ± 4
PANC-1	34 ± 3	94 ± 7	100 ± 5	97 ± 5	100 ± 3	91 ± 2	84 ± 1	80 ± 2	52 ± 13	92 ± 1	78 ± 3
A2780	30 ± 3	102 ± 12	96 ± 14	100 ± 3	106 ± 1	98 ± 2	101 ± 4	98 ± 0	40 ± 2	94 ± 5	5 ± 1
HeLa	35 ± 3	117 ± 14	108 ± 10	98 ± 9	93 ± 5	97 ± 8	91 ± 12	93 ± 9	28 ± 6	96 ± 10	11 ± 6
MCF-7	11 ± 1	98 ± 9	102 ± 6	95 ± 7	99 ± 5	96 ± 7	100 ± 3	91 ± 3	18 ± 2	102 ± 8	37 ± 3
SAOS-2	30 ± 8	98 ± 4	97 ± 7	102 ± 4	102 ± 5	103 ± 4	96 ± 7	95 ± 4	26 ± 3	103 ± 4	17 ± 5
MRC-5	29 ± 6	99 ± 12	97 ± 4	96 ± 9	108 ± 4	98 ± 4	92 ± 4	92 ± 6	39 ± 3	93 ± 4	29 ± 3

**Table 3:** IC_50_ values of pancracine and doxorubicin in human cancer and non-cancer cells ^a,b^.

Cell Type	Pancracine	Doxorubicin
Jurkat	5.07 ± 0.31	0.05 ± 0.02
MOLT-4	2.71 ± 0.25	<0.01
A549	2.29 ± 0.43	0.43 ± 0.06
HT-29	2.60 ± 0.51	0.77 ± 0.24
A2780	5.08 ± 0.43	<0.01
HeLa	5.03 ± 0.36	0.55 ± 0.05
MCF-7	2.68 ± 0.37	0.44 ± 0.10
SAOS-2	2.20 ± 0.25	0.1 ± 0.17
MRC-5	5.15 ± 0.34	0.72 ± 0.23

^a^ Results are expressed in µM. ^b^ Results are the mean values ± standard deviations of at least three independent replications.

## Supplementary Materials

The following are available online at https://www.mdpi.com/2223-7747/9/2/137/s1: Figure S1 GS/MS analysis of alkaloidal extract of Narcissus cv. Professor Einstein, Table S1 Alkaloids identified by GC/MS in fresh bulbs of Narcissus cv. Professor Einstein, Figure S2-1 ^1^H NMR spectra of 9-O-demethylmaritidine (**21**) in CDCl_3_, Figure S2-2 ^13^C NMR spectra of 9-*O*-demethylmaritidine (**21**) in CDCl_3_, Figure S3-1 ESI-HRMS spectra of new alkaloid 7-oxonorpluviine (**24**), Figure S3-2 ^1^H NMR spectra of new alkaloid 7-oxonorpluviine (**24**) in CD_3_OD, Figure S3-3 ^13^C NMR spectra of new alkaloid 7-oxonorpluviine (**24**) in CD_3_OD, Figure S3-4 gCOSY spectra of new alkaloid 7-oxonorpluviine (**24**) in CD_3_OD, Figure S3-5 gHSQC spectra of new alkaloid 7-oxonorpluviine (**24**) in CD_3_OD, Figure S3-6 gHMBC spectra of new alkaloid 7-oxonorpluviine (**24**) in CD_3_OD, Figure S3-7 NOESY spectra of new alkaloid 7-oxonorpluviine (**24**) in CD_3_OD.
