# Supplementary material for: Amaryllidaceae Alkaloids of Different Structural Types from Narcissus L. cv. Professor Einstein and Their Cytotoxic Activity
Source: Plants (Basel). 2020 Jan 22;9(2):137. doi: 10.3390/plants9020137 (PMC7076679; doi:10.3390/plants9020137)

Supplementary Material

Amaryllidaceae alkaloids of different structural types from *Narcissus* L. cv. Professor Einstein and their cytotoxic activity

Kateřina Breiterová[1], Darja Koutová[2], Jana Maříková[3], Radim Havelek[2], Jiří Kuneš[3], Martina Majorošová[2], Lubomír Opletal[1], Anna Hošťálková[1], Jaroslav Jenčo[1], Martina Řezáčová[2], Lucie Cahlíková[1]*

[1] ADINACO Research Group, Department of Pharmaceutical Botany, Faculty of Pharmacy, Charles University, Heyrovského 1203, 500 05 Hradec Králové, Czech Republic
[2] Department of Medical Biochemistry, Faculty of Medicine in Hradec Králové, Charles University, Šimkova 870, 500 03 Hradec Králové, Czech Republic
[3] Department of Organic and Bioorganic Chemistry, Faculty of Pharmacy, Charles University, Heyrovského 1203, 500 05 Hradec Králové, Czech Republic

Table of contents

Figure S1 GS/MS analysis of alkaloidal extract of *Narcissus* cv. Professor Einstein 2

Table S1 Alkaloids identified by GC/MS in fresh bulbs of *Narcissus* cv. Professor Einstein 3

Figure S2-1 ^{1}H NMR spectra of 9-*O*-demethylmaritidine (**21**) in $CDCl_3$ 4

Figure S2-2 ^{13}C NMR spectra of 9-*O*-demethylmaritidine (**21**) in $CDCl_3$ 4

Figure S3-1 ESI-HRMS spectra of new alkaloid 7-oxonorpluviine (**24**) 5

Figure S3-2 ^{1}H NMR spectra of new alkaloid 7-oxonorpluviine (**24**) in CD_3OD 5

Figure S3-3 ^{13}C NMR spectra of new alkaloid 7-oxonorpluviine (**24**) in CD_3OD 6

Figure S3-4 gCOSY spectra of new alkaloid 7-oxonorpluviine (**24**) in CD_3OD 6

Figure S3-5 gHSQC spectra of new alkaloid 7-oxonorpluviine (**24**) in CD_3OD 7

Figure S3-6 gHMBC spectra of new alkaloid 7-oxonorpluviine (**24**) in CD_3OD 7

Figure S3-7 NOESY spectra of new alkaloid 7-oxonorpluviine (**24**) in CD_3OD 8

Figure S1 GS/MS analysis of alkaloidal extract of *Narcissus* cv. Professor Einstein

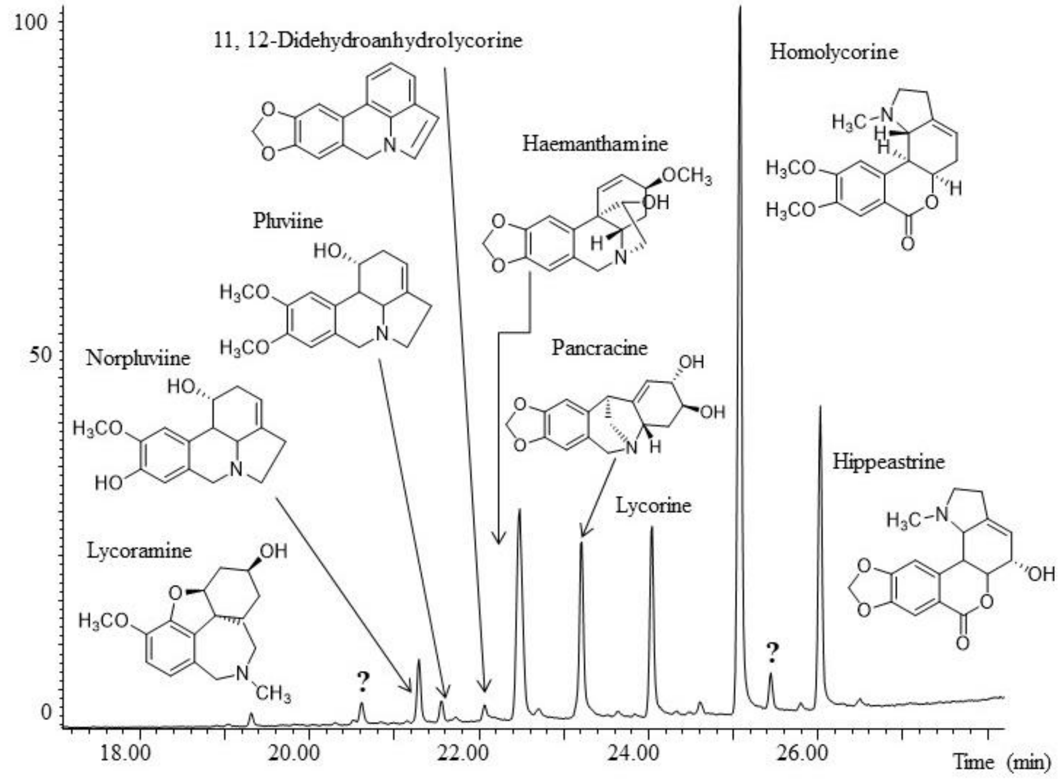

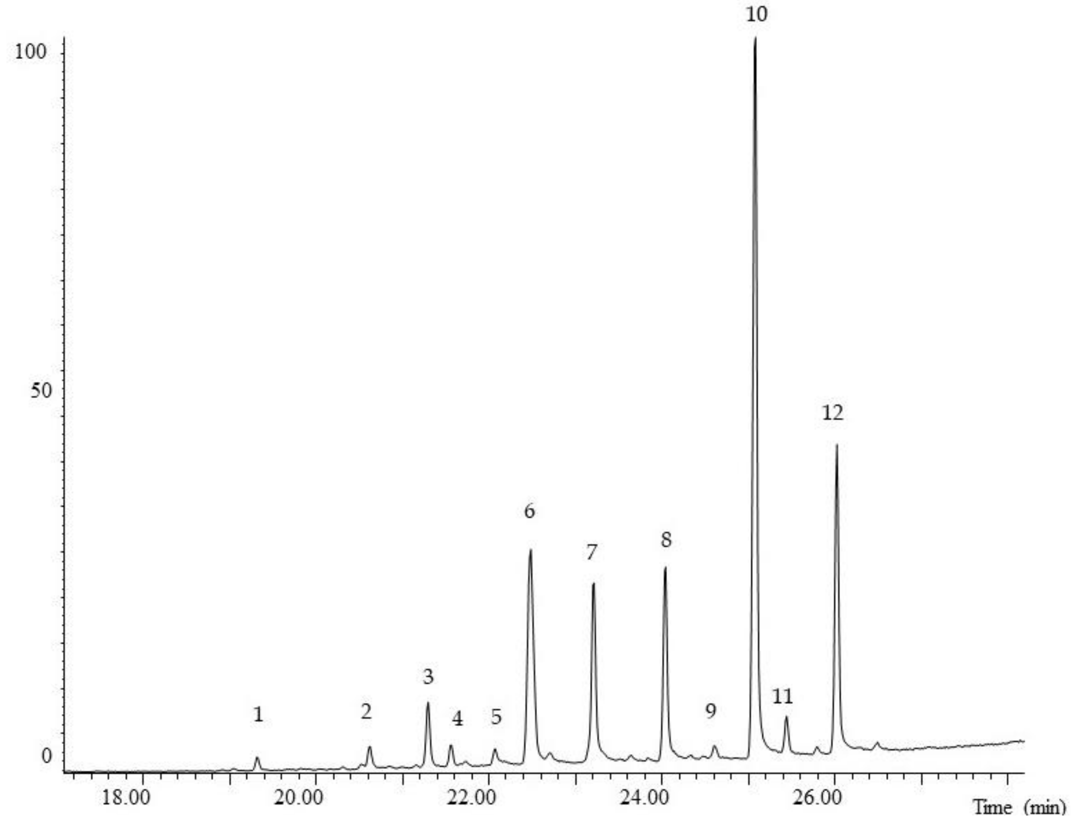

Table S1 Alkaloids identified by GC/MS in fresh bulbs of *Narcissus* cv. Professor Einstein

Compound	RT (min)	[M+] and characteristic ions, *m/z* (% of relative intensity)	%	Ref. for MS
1. Lycoramine	19.314	289(65), 288(100), 274(10), 232(10), 202(30)	tr	NIST 11
2. A1	20.625	?177(7), 109(100)	1	
3. Norpluviine	21.299	273(75), 273(33), 254(42), 242(6), 229(50), 228(100), 241(5)	3	NIST 11
4. Pluviine	21.569	287(81), 286(48), 268(52), 254(15), 243(66), 242(100), 228(23)	1	NIST 11
5. 11, 12– Didehydroanhydrolycorine	22.076	249(59), 248(100), 190(25), 163(7), 95(15)	1	[1]
6. Haemanthamine	22.486	301(15), 272(100), 240(15), 225(5), 211(15)	16	NIST 11
7. Pancracine	23.216	287(100), 286(20), 270(17), 243(17), 223(15), 214(16), 199(18), 185(26)	11	NIST 11
8. Lycorine	24.046	287(30), 268(24), 250(32), 227(68), 226(100), 211(5), 147(9)	10	NIST 11
9. 9–*O*-Methylpseudolycorine	24.604	303(35), 284(24), 243(86), 242(100)	<1	NIST 11
10. Homolycorine	25.086	301(-), 207(1), 178(3), 110(8), 109(100), 108(20), 94(3), 82(3)	39	[2]
11. A2	25.450	329(31), 269(76), 268(100), 250(93), 240(16), 226(81), 211(12), 147(15)	2	
12. Hippeastrine	26.033	315(-), 162(4), 134(4), 125(100), 96(36), 82(3)	15	[1]

1. Havlasová, J.; Šafratová, M.; Siatka, T.; Štěpánková, Š.; Novák, Z.; Ločárek, M.; Opletal, L.; Hrabinová, M.; Jun, D.; Benešová, N.; Kuneš, J.; Cahlíková, L. Chemical Composition of Bioactive Alkaloid Extracts from Some *Narcissus* Species and Varieties and their Biological Activity. *Nat. Prod. Commun.* **2014**, *9*, 1151-1155.
2. Cahlíková, L.; Ločárek, M.; Benešová, N.; Kučera, R.; Chlebek, J.; Novák, Z.; Opletal, L. Isolation and Cholinesterase Inhibitory Activity of *Narcissus* Extracts and Amaryllidaceae Alkaloids. *Nat. Prod. Commun.* **2013**, *8*, 781-785.

Figure S2-1 ^{1}H NMR spectra of 9-*O*-demethylmaritidine (**21**) in $CDCl_3$

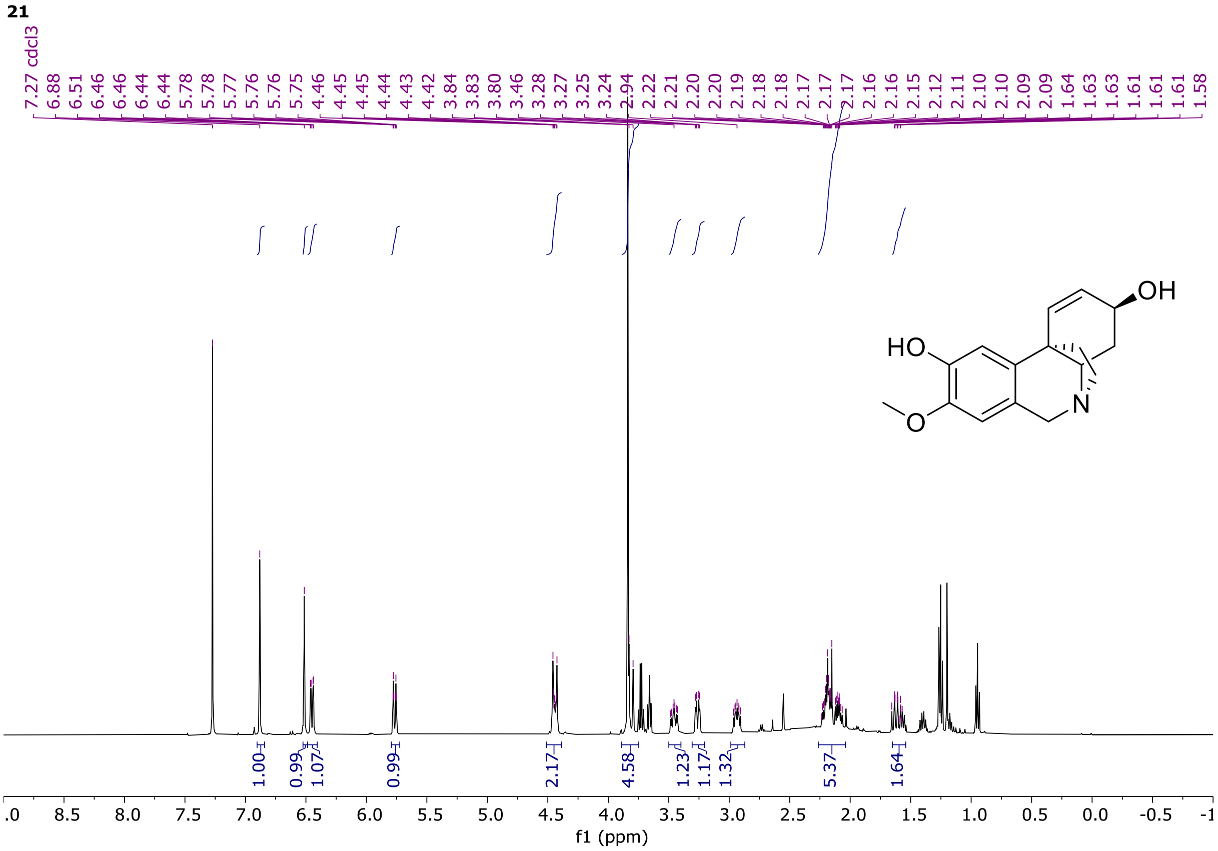

Figure S2-2 ^{13}C NMR spectra of 9-*O*-demethylmaritidine (**21**) in $CDCl_3$

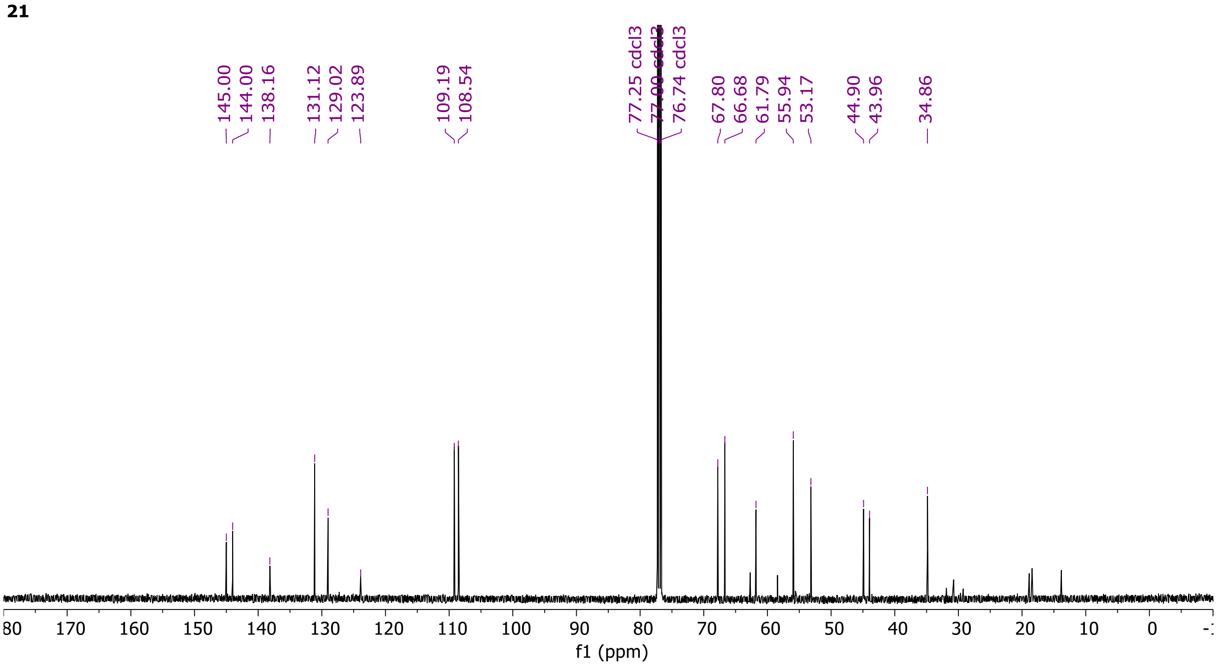

Figure S3-1 ESI-HRMS spectra of new alkaloid 7-oxonorpluviine (**24**)

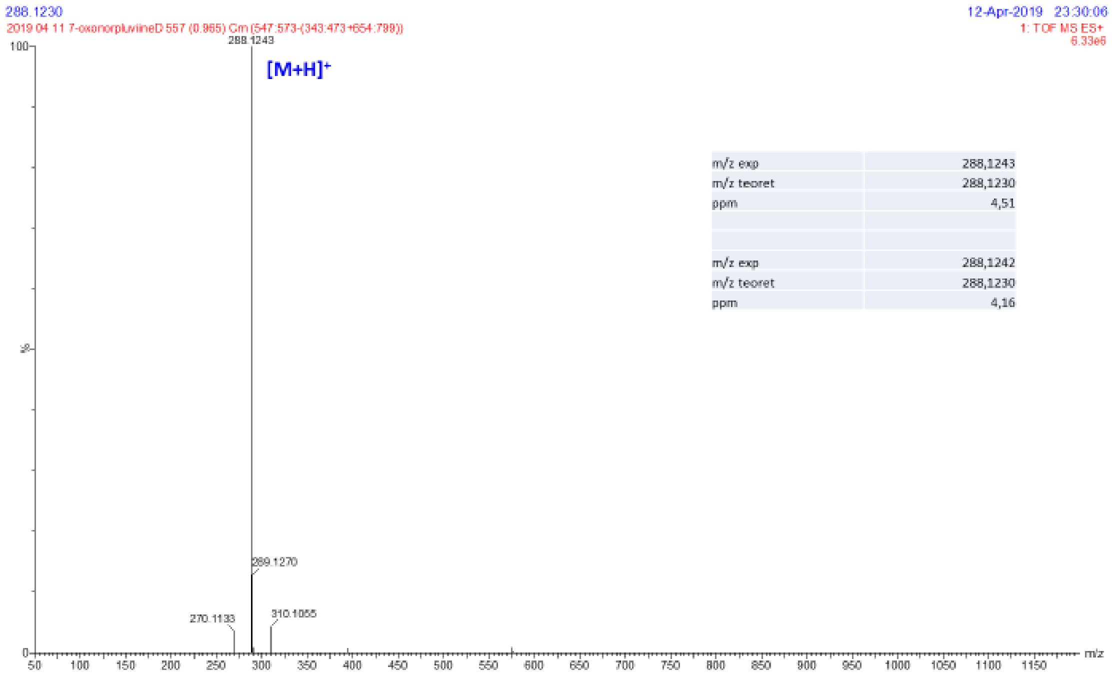

Figure S3-2 ^{1}H NMR spectra of new alkaloid 7-oxonorpluviine (**24**) in CD_3OD

24

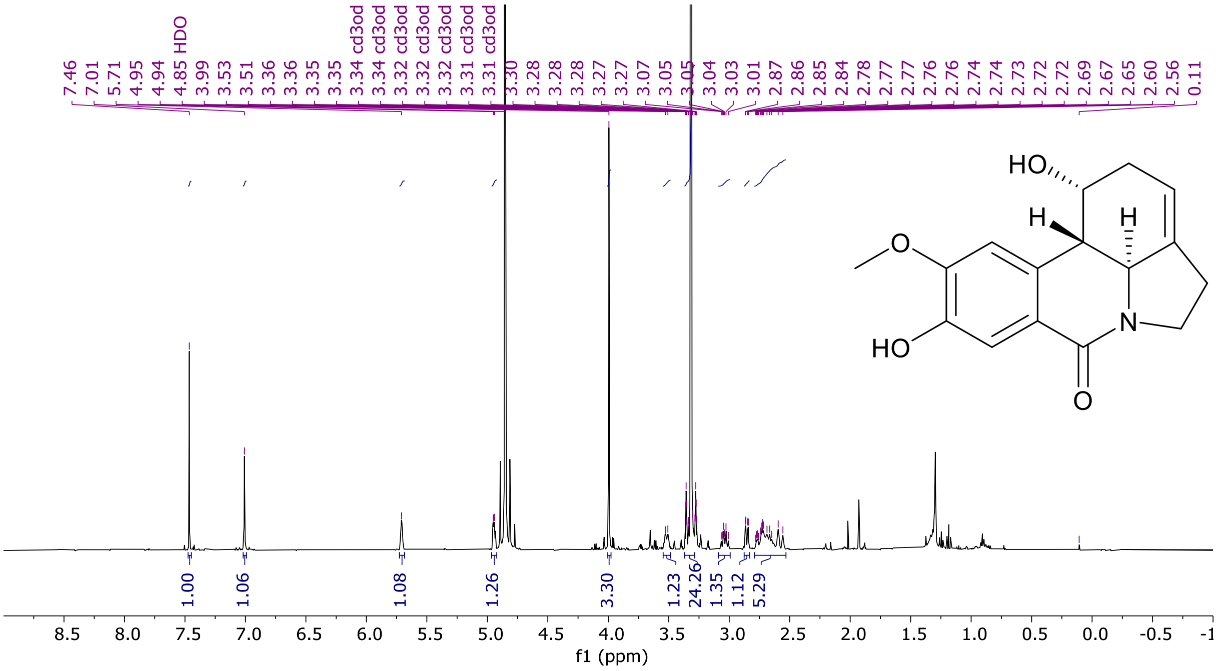

Figure S3-3 ^{13}C NMR spectra of new alkaloid 7-oxonorpluviine (**24**) in CD_3OD

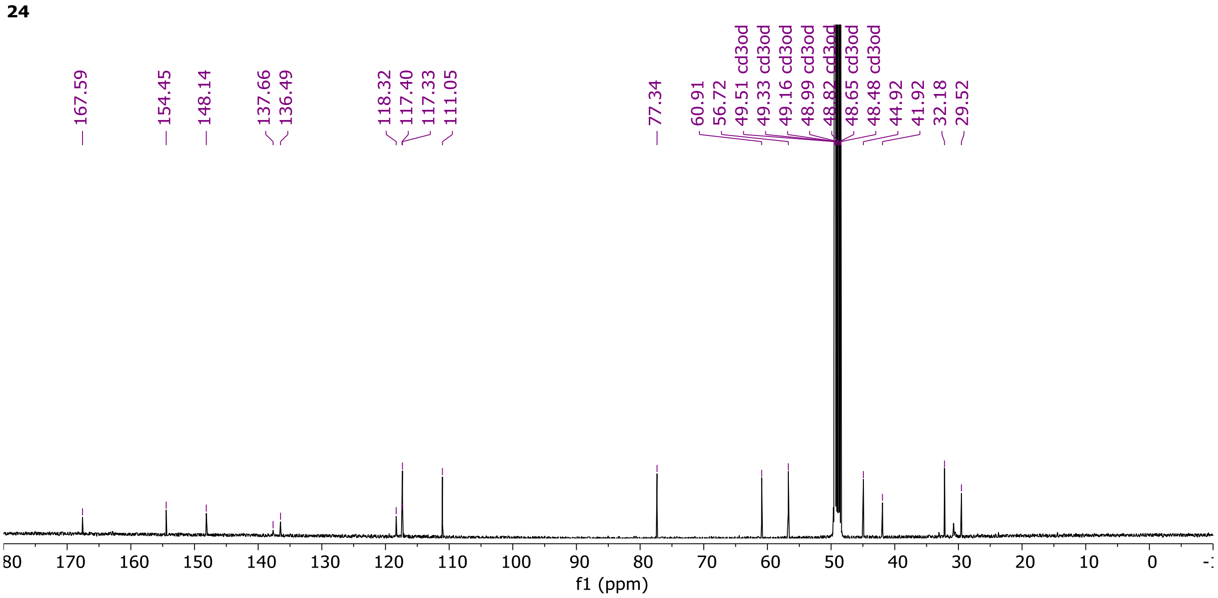

Figure S3-4 gCOSY spectra of new alkaloid 7-oxonorpluviine (**24**) in CD_3OD

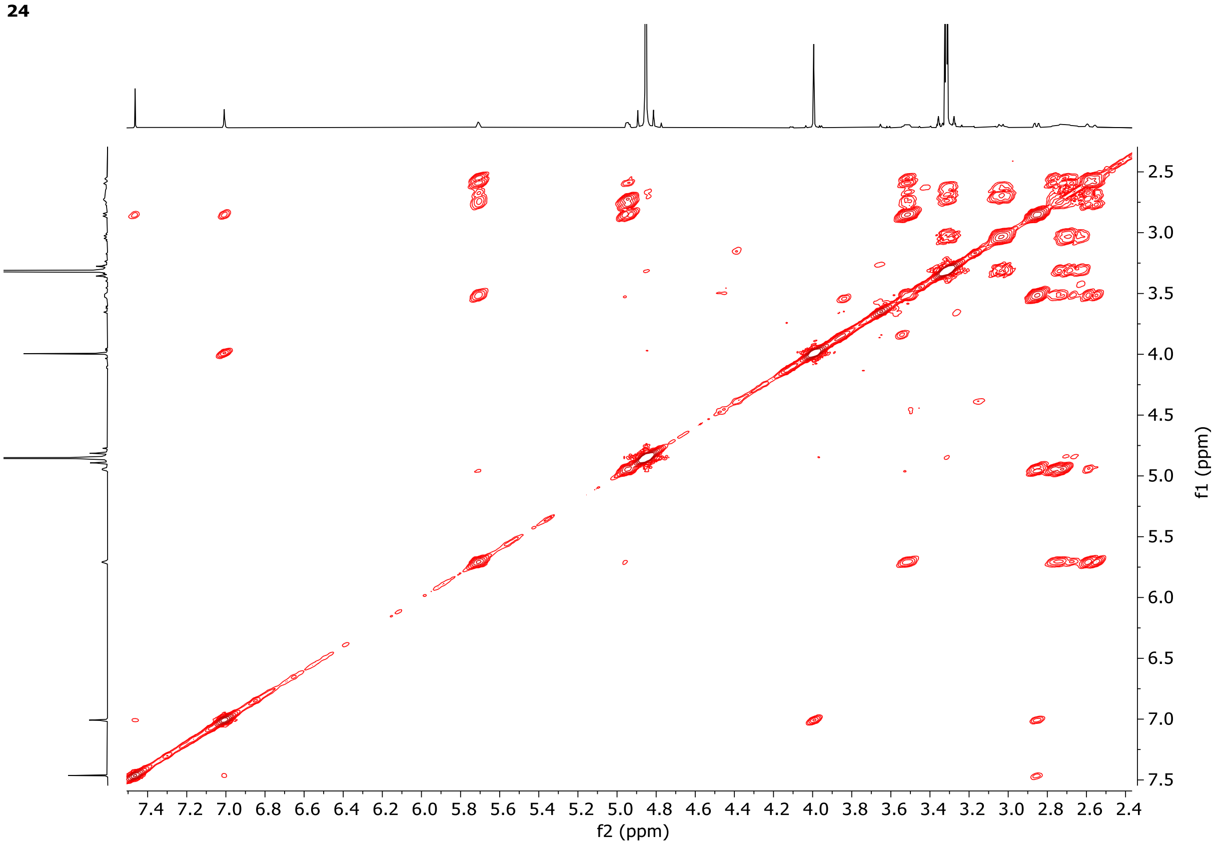

Figure S3-5 gHSQC spectra of new alkaloid 7-oxonorpluviine (**24**) in CD_3OD

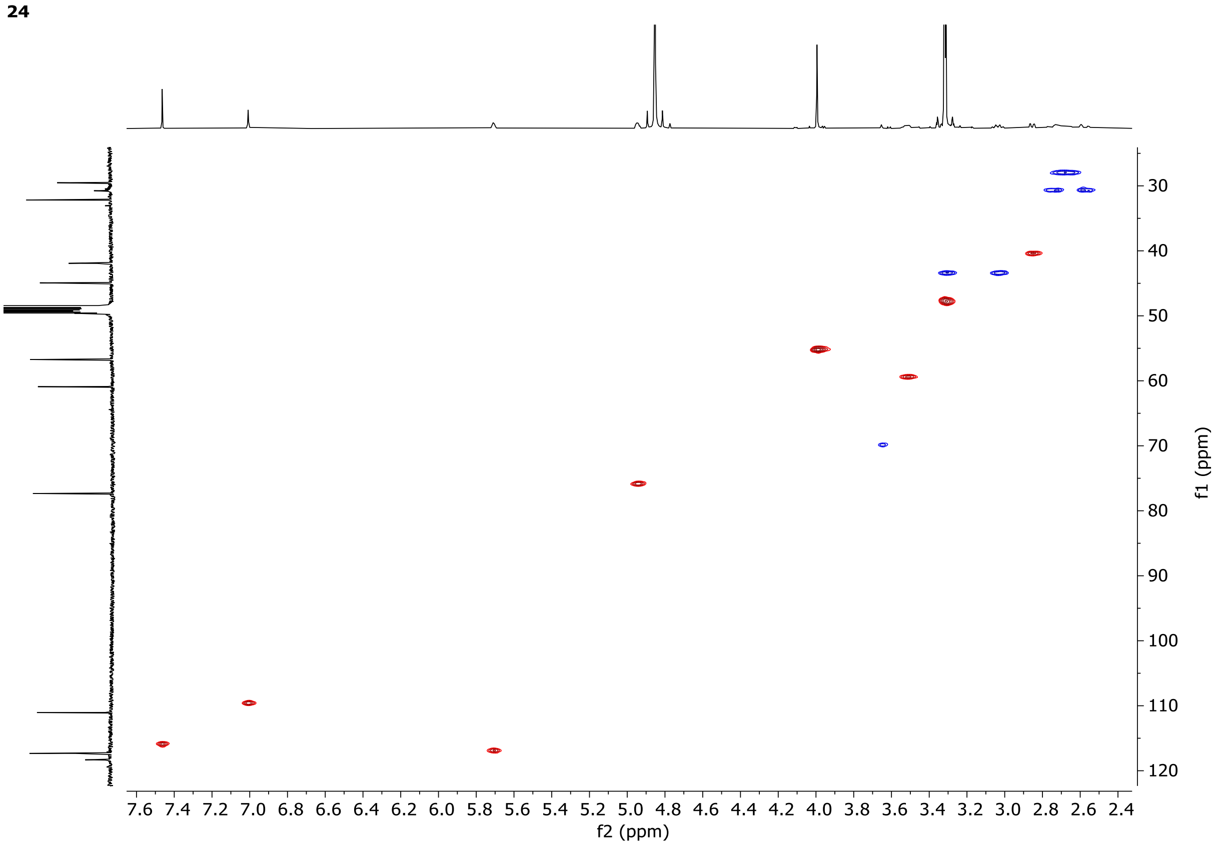

Figure S3-6 gHMBC spectra of new alkaloid 7-oxonorpluviine (**24**) in CD_3OD

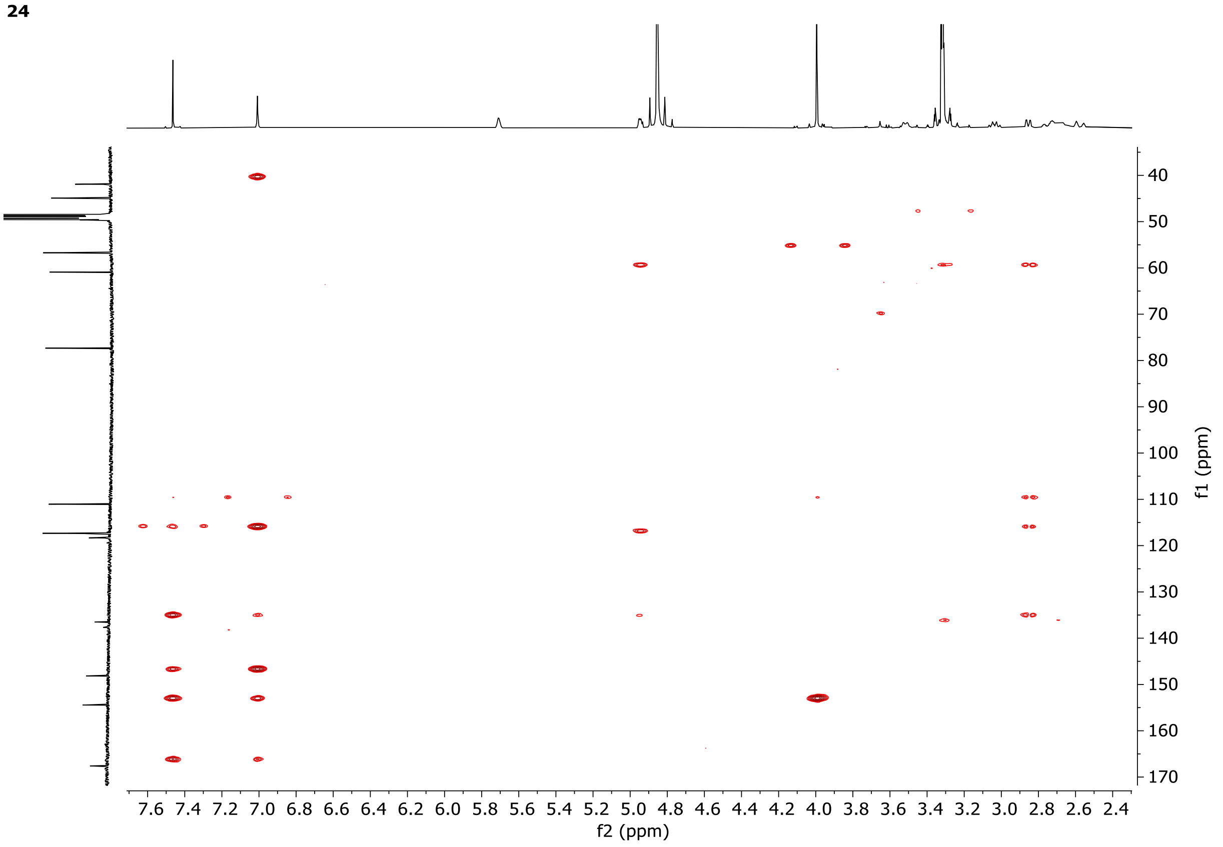

Figure S3-7 NOESY spectra of new alkaloid 7-oxonorpluviine (**24**) in CD_3OD

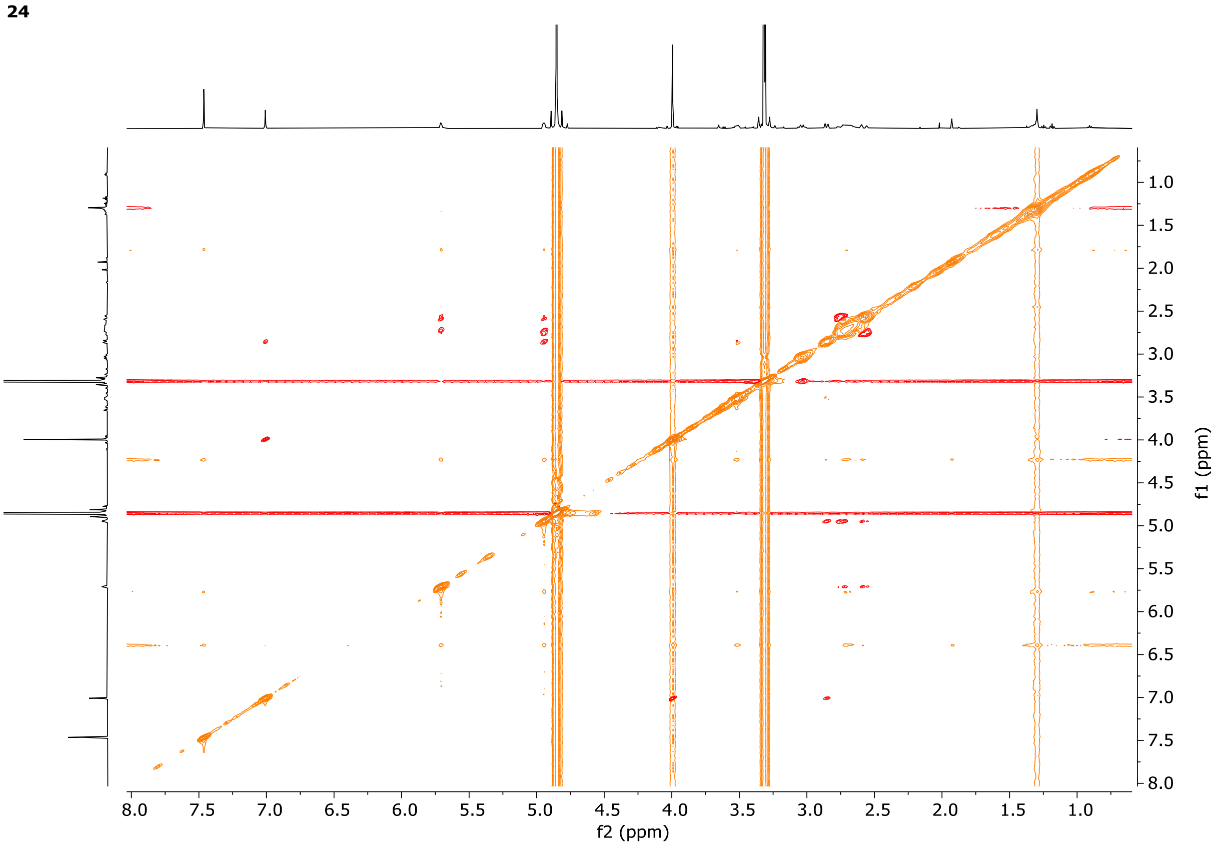

Supplement: Supplementary file 1 [file plants-09-00137-s001.pdf]
